# A Systematic Critical Appraisal of Non-Pharmacological Management of Rheumatoid Arthritis with Appraisal of Guidelines for Research and Evaluation II

**DOI:** 10.1371/journal.pone.0095369

**Published:** 2014-05-19

**Authors:** Lucie Brosseau, Prinon Rahman, Stéphane Poitras, Karine Toupin-April, Gail Paterson, Christine Smith, Judy King, Lynn Casimiro, Gino De Angelis, Laurianne Loew, Sabrina Cavallo, Jessica Mc Ewan

**Affiliations:** 1 School of Rehabilitation Sciences, Faculty of Health Sciences, University of Ottawa, Ottawa, Ontario, Canada; 2 Department of Community Health and Epidemiology, Dalhousie University, Halifax, Nova Scotia, Canada; 3 Centre for Global Health, Institute of Population Health, Ottawa, Ontario, Canada; 4 Department of Academic Affairs, Montfort Hospital, Ottawa, Ontario, Canada; 5 The Arthritis Society, Ottawa, Ontario, Canada; 6 University of Ottawa Library, Ottawa, Ontario, Canada; 7 École de Santé Publique, Université de Montréal, Montréal, Quebec, Canada; Universidad Peruana de Ciencias Aplicadas (UPC), Peru

## Abstract

Clinical practice guidelines (CPGs) have been developed to summarize evidence about the management of rheumatoid arthritis (RA) and facilitate the uptake of evidence-based knowledge by consumers, health professionals, health administrators and policy makers. The objectives of this review was to assess the quality of CPGS on non-pharmacological management of RA with a standardized and validated instrument - the Appraisal of Guidelines for Research and Evaluation (AGREE II) tool and summarize the key recommendations from these CPGs. Scientific literature databases from 2001 to 2013 were systematically searched and a total of 13 CPGs for RA was identified. Only a minority of AGREE II domains were effectively addressed by the CPGS. Scope and purpose was effectively addressed in 10 out of 13 CPGs, stakeholder involvement in 11 CPGs, rigor of development in 6 CPGs, clarity/presentation in 9 CPGs, editorial independence in 1 CPGs, and applicability in none of the CPGs. The overall quality of the included CPGs according to the 7-point AGREE II scoring system was 4.8±1.04. Patient education/self-management, aerobic, dynamic and stretching exercises were the commonly recommended for the non-pharmacological management of RA by the high-quality CPGs. The general clinical management recommendations tended to be similar among high-quality CPGs. Non-pharmacological management interventions were superficially addressed in more than half of the selected CPGs. CPGs creators should use the AGREE II criteria when developing guidelines. Innovative and effective methods of CPGs implementation to users are needed to ultimately enhance the quality of life of arthritic individuals. In addition, it was difficult to establish between strongly recommended, recommended and weakly recommended, as there is no consensus between the strength of the recommendations between the appraised CPGs.

## Introduction

Rheumatoid arthritis (RA) is an autoimmune pathology characterised by inflammation at the joints and in tissues surrounding other organs, which is usually accompanied by severe pain [1]. The prevalence of RA in the US in 2007 was about 1.5 million adults [2], and women were affected three times more than men [3]. The incidence is highly variable between nations, but is typically around 40 cases per 100,000 [4]. RA carries a great economic impact due to higher incidence in adults during their peak productivity years and the fact that long term treatment is required [5]. The estimated medical expenses for arthritis and rheumatism (excluding cost of time lost from paid or unpaid work) were estimated to be between $1.7 billion and $2.5 billion [6].

Evidence-based practice (EBP) is defined as the application and integration of the best available research evidence with clinical expertise and patient values by health care professionals delivering health care services [7]. There is a need for health professionals to include the best evidence-based practice (EBP) in order to provide optimal care to their patients [8]. EBP can include the use of clinical practice guidelines (CPGs) which are “systematically developed statements to guide the daily practice of health professionals about optimal health care for specific clinical circumstances” [9].

Health professionals working in rheumatology should appraise CPGs in their daily practice [10] in order to make the best informed decision about the optimal intervention for patient care. The Appraisal of Guidelines for Research and Evaluation II (AGREE II) (www.agreetrust.org) is an updated validated instrument that assesses the methodological quality of CPGs to ensure their high quality [11]–[13].

Numerous CPGs exist in the scientific literature, especially in the field of rheumatology. The CPG content can vary from a broad spectrum of recommendations including diagnosis, pharmacological and non-pharmacological management, surgery, multidisciplinary care, service delivery, or management of co-morbid conditions [14]. In the past, only one critical appraisal using AGREE I instrument has been conducted on CPGs available for the non-pharmacological management of RA [15]. Although the AGREE II instrument has been used to assess the quality of some CPGs for the management of osteoarthritis [10], [16]–[17], the quality of non-pharmacological CPGs for the management of RA have not been assessed with this updated instrument. Therefore, there is a need to appraise non-pharmacological CPGs for RA – so that health care providers can identify which CPGs in RA are high-quality and reliable to use in their practice. To our knowledge, no publications have used AGREE II to appraise CPGs for RA before this paper. The objectives of this critical appraisal review were: 1) to systematically identify CPGs for the non-pharmacological management of RA found in the scientific literature for use by multidisciplinary healthcare providers; 2) assess the quality of the selected CPGs using the AGREE II instrument and 3) identify and compare the non-pharmacological recommendations from the selected CPGs.

## Methodology

The PRISMA statement [18] was used to report this systematic review (Checklist S1) and the Cochrane methodology (www.cochrane.org) [19] was used to identify, select and analyze the data.

### Systematic Search and Selection of CPGs

A librarian (JM) used specific key words to complete a systematic literature search (Appendix S1) in the following databases: AMED, CINHAL, Medline, and Embase (Figure S1). A hand search was also performed with existing guideline inventories such as PEDro (http://www.pedro.fhs.usyd.edu.au/index.html) [20], the National Guideline Clearinghouse (http://www.guideline.gov/) [21], Guideline International Network (http://www.g-i-n.net/) [22], Turning Research into Practice (TRIP) (http://www.tripdatabase.com/) [23] and by searching the reference lists of the selected CPGs. Two trained research assistants followed the selection criteria described in appendix S2, to identify and select CPGs that considered non-pharmacological management of RA for quality appraisal. CPGs which contained at least one recommendation related to the non-pharmacological management of RA, used a systematic review to identify and grading systems to evaluate the evidence were included. In addition, CPGs that were published before 2001 and written in a language other than English were not considered. Ethics approval was not required as this is a systematic literature review.

### Evaluation of the CPG Quality

The AGREE II instrument is a CPG appraisal instrument consisting of 23 items across six domains: 1) scope and purpose; 2) stakeholder involvement; 3) rigour of development; 4) clarity and presentation; 5) applicability; and 6) editorial independence. Each item is scored with a 7-point scoring system. If a CPG fulfilled none of the criteria for an item, it is graded with a 1, but if all the criteria are met and presented in good quality a grade of 7 is assigned. The purpose of this instrument is to provide a framework to: 1) assess the quality of guidelines; 2) provide a methodological strategy for the development of guidelines; and 3) provide what information should be reported in the guidelines (http://agree.machealth.ca/players/open/index.html) [24].

Three evaluators were trained over two months with the AGREE II instrument using the tutorial found on its website (http://agree.machealth.ca/players/open/index.html) [24]. A senior research assistant with experience with AGREE II, one of the instrument’s developers, and the director of the Cochrane Effective Practice and Organization of Care and Cochrane Collaboration group were available to provide assistance with evaluating the CPGs when necessary.

Two pairs of experienced evaluators independently assessed the selected CPGs with the AGREE II instrument and to prevent a potential information bias a third experienced evaluator was involved when a co-author of the present review was also a co-creator of an included CPG. A standardised electronic form found on the AGREE II website was used to compile the data (www.agreetrust.org) [11]. A score between one and seven was assigned depending on whether the items met the criteria or considerations, and the completeness and quality of reporting. Some of the AGREE II items were not relevant to the guidelines assessed and because AGREE II does not include a ‘not applicable’ response item in the scale these items was given a score of one. After completing the appraisal of all 23 items, each appraiser provided an overall quality assessment score out of 7 on the CPG and stated whether they 1) recommend the CPG for practice 2) recommend the CPG for practice but with modifications or 3) do not recommend the CPG for practice (p. 10 http://www.agreetrust.org/wp-content/uploads/2013/06/AGREE_II_Users_Manual_and_23-item_Instrument_ENGLISH.pdf). According to the AGREE II user manual (www.agreetrust.org), the overall quality assessment requires the user to make a judgment about the quality of the CPG, taking into account that the potential bias of the guidelines has been adequately addressed and the recommendations are both internally and externally valid and feasible for practice [12]–[13].

### Non-pharmacological Interventions of RA

In addition to assessing the methodological quality, all the non-pharmacological interventions assessed in the included CPGs were compiled with the strength of the recommendations (Table S1). Standardised categories were created for equal comparison of the strength of recommendations across CPGs. They were: 1) strongly recommended; 2) recommended; 3) weak evidence; and 4) insufficient evidence (Table S1). The categories took into consideration that CPGs had different scoring systems such that any recommendation was put in an equivalent category than it had in the original CPG. A “strongly recommended” grade was assigned to the highest CPG grading that would include one or more high-quality randomised controlled trials (RCT). A “recommended” grade was assigned when a grading had one or more controlled clinical trials (CCTs) or a lower quality RCT; this grade signifies that the intervention is still reliable to guide practice most of the time. Strong clinical or expert opinions founded on current practice are graded as “weak evidence” and should be applied with caution. If scientific evidence is lacking or there are conflicting results that have not been supported by clinical or expert opinion, it was given a grade of “insufficient evidence”.

### Data Analysis

The AGREE II instrument was used for the broad appraisal of the quality of CPGs. The quality score of each CPG was calculated for each of the AGREE II domains. Domain scores were calculated by summing up all the scores of the individual items and calculating the total as a percentage of the maximum possible score for that domain.

The scaled domain score:

(Obtained score- Minimum possible score)/(Maximum possible score).

Maximum possible score = 7 (strongly agree)×3 (items)×2 (appraisers).

Minimum possible score = 1 (strongly disagree)×3 (items)×2 (appraisers).

The scaled domain score were obtained by calculating the mean of the 2 evaluators’ scores as shown in the AGREE II user manual (www.agreetrust.org) [11]. The two evaluators used the concordance calculator developed by McMaster University (http://fhswedge.csu.mcmaster.ca/cepftp/qasite/AGREEIIRaterConcordanceCalculator.html) [25] to obtain accurate quality ratings for each domain of the AGREE II criteria Items scores were discussed by the two reviewers when large scoring discrepancies (≤3 points difference in the score assigned by the appraisers to the same item) or if the individual item scores of the two reviewers are greater than or equal 1.5SD from the mean for each of the domain. The purpose of the discussion was to resolve the discrepancies in scoring by consensus between the two raters.

### Identifying High Quality Guidelines

The AGREE II consortium has not set a minimum or maximum range for the domain quality and this makes it difficult to distinguish between high and low CPGs. Therefore the criteria of previous critical appraisals using the AGREE I [10], [15], [26]–[28] were used with the updated AGREE II instrument to identify and recommend high quality guidelines. Domain scores ≥60% is considered effectively addressed. A guideline is considered high quality and recommended if it effectively targets at least 3 out of the 6 domains including rigour of development (domain 3).

### Inter-rater Reliability Study

To ensure a high inter-rater reliability between the two appraisers who had equal training, an intra-class coefficient (ICC) based on ANOVA was calculated in SPSS (Version 20) [28]. The sub-total for the item scores in each domain obtained by one evaluator were compared with those rated by the second evaluator. This statistical procedure was performed after the consensus process explained above.

### Descriptive Analysis

A scatterplot was conducted on excel 2013 plotting the overall quality assessment scores of each included CPG and the year it was published. This additional analysis was conducted to ascertain if the quality of CPGs improved over time.

## Results

Of the 1136 citations systematically searched, duplicates were eliminated leaving 827 residual citations for title and abstract review (Figure S1). Based on the title and abstract, 811 citations were subsequently excluded because they did not address a CPG related to non-pharmacological management of RA and twelve CPGs were included (Figure S1). One additional CPG was identified and included after consulting the reference list of the included publications (Figure S1).

Based on the selection criteria (Appendix S2) a total of thirteen CPGs on the non-pharmacological management of RA [30]–[44] were retrieved by the two trained evaluators (Figure S1).

Six CPGs were developed for the early stage RA (first two years from the onset) [30]–[31], [33], [35], [43], [44] and the residual CPGs (n = 7) were more general in terms of the evolution of RA [31], [33], [35]–[39]. Three separate guidelines and an accompanying online guideline aid [41] were developed by the Ottawa Panel, but as the guideline development process was identical for all three guidelines they were evaluated as one. The 13 CPGs can be divided into two categories: general management [30]–[35], [37], [41]–[42] and non-pharmacological management of RA [34]–[36], [38]–[42] (see Table 1).

**Table 1:** Guidelines that considered pharmacological and non-pharmacological interventions.

Guidelines that considered pharmacological+non-pharmacological interventions	Guidelines that only considered non-pharmacological interventions.
**ACR [30]**	Forrestier et al. [34]
**BSR [31]**	Gossec et al. [35]
**BSR [32]**	Hurkman et al. [36]
**EULAR [33]**	Ottawa Panel [38]–[42]
**NICE [37]**	
**RACGP [43]**	
**SIGN [44]**	

ACR: American College of Rheumatology, BSR: British society of rheumatology; EULAR: The European League against rheumatism; NICE: National Institute for health and Clinical Excellence; SIGN: Scottish Intercollegiate Guidelines Network.

### Evaluation of Selected CPGs

The quality assessments of the CPGs based on the AGREE II scores are presented in Table 2 and Appendix S3 presents the individual item scores for each domain graded by the two evaluators for the six domains. Based on the quality scores from the AGREE II, 6 [34], [36], [38]–[42] out of the 13 CPGs are considered high quality CPGs. All six CPGs adequately addressed (≥60%) domain 3: rigour of development and 2 or more additional domains. The overall quality for these six CPGs ranged from 5 to 6.5 out of 7 (see table 2). The majority of the CPGs effectively addressed domain 1, 2 and 4 with a mean quality score of 70% ±42%, 71% ±26% and 64.0% ±28% respectively. Conversely, domain 3, 5 and 6 were poorly addressed by the majority of the guidelines. All three domains had a low mean quality score (<60%) of 52.4%±23%, 18.5%±35% and 38.3%±23%.

**Table 2:** Quality Scores using AGREE II Instruments for included CPGs on RA.

Agree II domains	ACR	BSR	BSR &BHP	Eular	Forestieret al.	Gossecet al.	Hurkmanet al.	NICE	OttawaPanel	RACGP	SIGN	(mn ±SD)
	[30]	[31]	[32]	[33]	[34]	[35]	[36]	[37]	[38]–[42]	[43]	[44]	
Domain 1	17%	83%	89%	17%	86%	89%	47%	75%	97%	97%	81%	70.4%±42%
Domain 2	25%	75%	75%	36%	67%	64%	86%	89%	72%	86%	94%	70.6%±26%
Domain 3	11%	34%	32%	44%	71%	58%	69%	52%	65%	84%	55%	52.4%±23%
Domain 4	11%	83%	67%	14%	100%	39%	94%	39%	61%	100%	97%	64.0%±28%
Domain 5	17%	50%	8%	0%	46%	0%	4%	17%	10%	17%	35%	18.5%±35%
Domain 6	38%	38%	46%	0%	50%	21%	8%	92%	42%	33%	54%	38.3%±23%
Quality of CPGs (mn±SD)	3.5±0.5	4.5±0.71	4.5±0.71	4±1.41	5.5±0.5	5±0	5±0	4±0	5.5±0.71	6.5±0.71	5±1.41	4.8±0.61
Assessed by	PR & LB	PR &LB	PR & LB	PR & LB	PR & LB	PR & LB	PR & LB	PR & LB	PR & KTA	PR & LB	PR & LB	PR & LB

ACR: American College of Rheumatology, BSR: British society of rheumatology; EULAR: The European League against rheumatism; NICE: National Institute for health and Clinical Excellence; SIGN: Scottish Intercollegiate Guidelines Network; 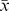: mean; SD: standard deviation.

According to the number of domains effectively addressed (Table 2) and overall assessment (Appendix S3), 6 CPGs [34], [36], [38]–[42,36] are recommended, 6 CPGs [30]–[32], [35], [37], [44] are recommended but with modifications and 1 CPG [33] is not recommended for practice by the evaluators.

### Non-pharmacological Recommendations for the Management of RA

Table S1 presents specific non-pharmacological recommendations cited in the selected CPGs for the management of RA. The strength of the recommendations for each intervention was provided by the developers of the included CPGs [30]–[44] including the People Getting a Grip on Arthritis Program (PGRIP) [42] based on the Ottawa Panel CPGs [39]–[41] is also provided. The recommendations formulated by these CPGs were categorized by alphabetical order: 1) electrotherapy; 2) other intervention; 3) team approaches; 4) therapeutic exercises; and 5) weight management. Generally speaking, the strength of the recommendations varied largely amongst the 13 CPGs (Table S1). All CPGs failed to mention whether these non-pharmacological recommendations were contraindicated or represented harms in the management of RA.

#### Electrotherapy

CPGs which considered electrotherapy for RA focus on electrical stimulation of muscle, TENS, Low Intensity Laser Therapy (LILT) and therapeutic ultrasound. Large discrepancies on the strength of recommendations existed for TENS, LILT and therapeutic ultrasound. For these particular electrotherapeutic modalities, the recommendations ranged from insufficient evidence to a strong recommended. Amongst the thirteen CPGs, four CPGs [36], [38]–[42] found insufficient evidence to recommend electrical stimulation of muscle. Both high and low frequency TENS were recommended by the NICE guidelines and strongly recommended by the Ottawa Panel Guidelines [37], [41]. The Ottawa Panel guideline was the only non-pharmacological CPGs which strongly recommended TENS, Low Level Laser Therapy and therapeutic ultrasound. The Remaining nine CPGs either found insufficient evidence to recommend any of the four modalities in electrotherapy or these modalities were not applicable to the CPG.

#### Other therapies

A larger variety of other interventions were also assessed. These non-pharmacological interventions included acupuncture, assistive devices, balneotherapy, complementary and alternative therapies, energy conservation, foot orthoses and insoles, heat therapy, cryotherapy, hydrotherapy, joint protection, paraffin wax application combined with or without exercises, patient education and splinting.

The 13 CPGs [30]–[44] recognised that acupuncture, assistive devices, heat therapy/cryotherapy provided either insufficient evidence or was weakly recommended. Balneotherapy [33], complementary and alternative therapies [43], energy conservation [30], hydrotherapy [33], and paraffin wax application alone [37], Paraffin wax application combined with or without exercises is either recommended or strongly recommended by 1 CPG [41].

Six out of the thirteen CPGs either recommended or strongly recommended “joint protection and splinting” [30], [32], [36]–[43] and foot orthoses and insoles were recommended by four CPGs [31], [35], [40], [44]. Joint protection was recommended or strongly recommended by seven CPGs [30], [32], [40], [43]. Patient education/self-management was strongly recommended or recommended by 10 CPGs [30]–[34], [36]–[42] and the remaining 3 CPGs [35], [43]–[44] found weak to insufficient evidence. Splinting was recommended or strongly recommended by two CPGs [39], [45].

#### Team approach

All CPGs [30]–[34], [37], [44] that assessed Multidisciplinary Team Approach in the management of RA unanimously recommended its use. These CPGs alternatively either weakly recommended or recommended the representation of several professions to be part of the team (i.e. Nutrition, Medicine, Nursing, Occupational Therapy, Podiatry/Chiropodiatry, Pharmacy, Physiotherapy, Psychology, Social Work).

#### Therapeutic exercises

Aerobic exercises was either recommended or strongly recommended by 12 of the 13 CPGs [30]–[42], [44] and dynamic exercises were strongly recommended or recommended by all 13 [30]–[44] CPGs. Strengthening exercises and whole body exercises were recommended by 12 [30]–[42], [44]. Stretching was cited and recommended by only one CPG [37]. Two CPGs [38], [44,] strongly recommended specific low intensity exercises in the management of RA.

#### Weight management

There was a large variation regarding the strength of recommendations for weight management in RA for the 6 CPGs that assessed this intervention [33]–[35], [37], [43]–[44]. One CPG [43] either strongly recommended or recommended diet, diet supplement and diet combined with physical activity, diet supplement and diet alone for the management of RA. Another CPG [44] found evidence to recommend control of weight and diet for weight management.

### Inter-rater Reliability Study

The AGREE II scores exhibited an overall very good inter-rater reliability with an ICCs values ranging from 0.91 to 0.95 (high reliability) depending on the domain assessed (Table 3). The high ICC scores confirm that the results obtained between the two evaluators are highly reliable (Table 3).

**Table 3:** Inter rater reliability study results for included CPGs a.

Agree II Domain	P(G)MS^b^	RMS	EMS	*n*	K	ICC (Random)	Lower 95% Cl	Upper 95% Cl	P value
Domain 1	54.382	.045	.545	12	2	.990 (high)	.963	.997	.000
Domain 2	34.778	3.200	1.422	12	2	.959 (high)	.835	.990	.000
Domain 3	165.756	16.200	.494	12	2	.987(high)	.947	.997	.000
Domain 4	58.482	3.682	5.082	12	2	.913(high)	.677	.977	.000
Domain 5	35.64	.182	.482	12	2	.986(high)	.950	.996	.000
Domain 6	21.467	.200	.533	12	2	.975(high)	.900	.994	.000

a CP Gs =  clinical practice guidelines; P(G)MS = Patients’ (Guideline’s) Mean Square; RMS =  Rater’s Mean Square; EMS =  Error Mean Square; n =  sample size; K =  number of measurements; ICC =  intraclass correlation coefficient; CI =  confidence interval.

b Temporary PMS, RMS, and EMS value.

### Descriptive Analysis

Based on the overall quality scores out of 7 (figure S2) there is an improvement from the earliest published CPG ACR [30] to the CPGs published 11 years later such as Hurkman et al. 2011 [36], Ottawa Panel 2011 [40] and SIGN 2011 [44]. However the results from figure S2 illustrates that the quality of CPGs does not improve overtime, Eular (2007) [33] and NICE (2009) [37] received lower quality scores compared to Ottawa Panel [2004] guidelines which was published earlier in 2004.

## Discussion

This systematic review identified a total of 13 CPGs, of which 7 were more general and comprised a larger proportion of pharmacological compared to non-pharmacological recommendations [30]–[33], [37], [43]–[44]. Only 6 CPGs were entirely devoted to non-pharmacological interventions for RA [34]–[36], [38]–[43]. Based on the AGREE II quality scoring instrument 6 CPGs [34], [36], [38]–[43] were considered high quality CPGs (adequately addressed rigor of development >60% in addition to addressing two or more domains effectively) and recommended for the non-pharmacological management of RA and the remaining CPGs were recommended for practice but with modifications. The majority of the 13 CPGs [30]–[44] recommended that non-pharmacological interventions be combined with pharmacological interventions in the global management of RA. Only two CPGs distinguish specific recommendations for the management of RA during the first two years since onset [31], [34] and after the two first years [32]. Foot orthosis/insoles, joint protection, patient education/self-management, multidisciplinary team approach, aerobic, dynamic, stretching, strengthening and whole body exercises were the common non-pharmacological interventions recommended by majority of the selected CPGs.

### Quality Scores of CPGs

None of the CPGs were found to adequately address all six of the domains when using the AGREE II appraisal instrument. The majority of the included CPGs obtained a high AGREE II quality score for domain 1 (scope and purpose), domain 2 (stakeholder involvement) and domain 4 (clarity of presentation). The scope and purpose were well addressed in 10 [31]–[32], [34]–[35], [37]–[44] out of the 13 CPGs and the 3 [30], [33], [36] CPGs that were not able to effectively target this domain lacked information on the target population [36] and the clinical question addressed by the CPG [30], [33], [36]. Eleven [31]–[32], [34]–[44] out of the 13 CPGs effectively addressed stakeholder involvement. However, 2 CPGs [30], [33] received a score <60% due to insufficient information on how the views and preferences of the target population were sought and the target users of the CPGs. While all the CPGs considered the views and preferences of patient and patient representatives, most CPGs lacked information on the methods by which the views and preferences were sought and how these outcomes impacted the development and formation of the recommendation. Generally most of the CPGs received high scores for clarity of presentation (domain 4), however 4 CPGs [30], [33], [35], [37] were not able to effectively target this domain, because the recommendations presented were unclear especially regarding the duration and dosage for non-pharmacological interventions.

Domain 3 (rigour of development) obtained a relatively low mean AGREE II score for the 13 CPGS and 6 CPGs effectively targeted (≥60%) this domain [34], [36], [38]–[43]. Special attention should be given to the rigor of development when examining the quality of the results [26], as CPGs were considered high quality if the rigor of development was effectively targeted (>60%) in addition to targeting 2 or more other domains. Receiving a high score for this domain indicated minimum bias and evidence based design in the development of the CPGs. All 13 CPGs [30]–[44] reported the relevant data bases used and explicit link between the recommendations and supporting evidence. The majority of the CPGs were unsuccessful in targeting this domain due to insufficient information on the inclusion and exclusion criteria used [30]–[31], [34], [36], [43], the methods for formulating the recommendations [30]–[32], [35], [37], [44] and an explicit statement about when and what method will be used to update the guideline [30]–[33], [35]. Although most CPGs provided the benefits when considering non-pharmacological recommendations, insufficient information about the risks and side effects for a weak evidence recommendation was provided. This might be due to the fact that RCTs on non-pharmacological interventions rarely reported side effects (i.e harm) and risks. “Harm” is difficult to assess when developing a CPG, as it is based on the RCT which studies the effectiveness. As a result some CPGs [30]–[32], [35] received low scores for this domain because the information was not available to the evaluators. It is unrealistic to expect busy clinicians to search for required or supplementary information; therefore CPGs should be reported in sufficient details with the additional information included and easily accessible.

Domain 5 (Applicability) obtained the lowest mean AGREE II ratings for all 13 CPGs and none were able to effectively address this domain. A rational for such low scores can be attributed to the criteria for this domain. In order for CPGs to receive high ratings, it should report the tools for application, the barriers and cost in applying the recommendations, and monitoring and auditing criteria. It might be premature to use these criteria to assess the applicability of the CPGs, because the developers usually focus on planning the dissemination and implementation of the CPGs [24]. The inclusion of the “applicability” domain is controversial, as the development of a CPG takes considerable time with extensive human and financial resources. Applicability requires well conducted RCTs and cluster RCTs in order to be properly implemented [45] and should ideally include an economic evaluation. The Knowledge-To-Action Cycle [46] should be taken into account when conducting an implementation study. These missing items and the prematurely assessed domain could directly affect the evaluation of the overall quality of the CPGs included in this synthesis paper. AGREE II should be applied in two phases: 1) the development phase and 2) the implementation phase.

Domain 6 (editorial independence) also received a low mean quality score for the 13 CPGs [30]–[44] and only one CPG [37] effectively targeted this domain. The scores for this domain was low, because several CPGs failed to mention the information related to conflict of interest [31]–[32] in their publication and whether the views of the funding body have influenced the content of the guideline [30]–[32], [34]–[35].

The quality scores for the CPGs in the review are important to consider and are useful when making decisions as to recommend a specific CPG. The criteria from previous critical appraisals with AGREE I are used with the AGREE II instrument to identify high quality guidelines in this systematic reviews. While the new AGREE II instrument does not provide a threshold to identify “high” quality CPGs from “low” CPGs, the domain scores are helpful for comparing guidelines [26]. CPG developers should use the AGREE II criteria (www.agreetrust.org) [11] when developing their CPG in order to publish higher quality CPGs [10], [15].

The results of this review can be partially compared to the only known systematic review done for the quality appraisal done of CPGs on the use of physiotherapy in RA with AGREE I [15]. Although the same CPGs were considered in both reviews, the present review used the updated AGREE tool (AGREE II) to assess the CPG quality. The findings of this review agree with the results of Hurkman et al [15], where none of the CPGs for both reviews effectively targeted all six of the domains. Both reviews found that rigor of development and applicability were not well addressed by most of the CPGs and finally for both reviews the domain scores ranged from >70% for domain 1 (scope and purpose) to <50% for domain 5 (applicability).

### Strength of Non-pharmacological Recommendations

Surprisingly the strength of the recommendations differed among the CPGs. Part of the reason for this discrepancy could be attributed to the difference in the years of the CPGs developed. However, recently published CPGs based on the same available literature also differed in strength for the recommendations. A reason for this might be due to the method of analysis in order to ensure consensus about the strength of the recommendations for each non-pharmacological intervention.

The strength of recommendations may also vary according to the stages of RA [31]–[32]. Furthermore, non-pharmacological management of RA depends on the stage and progression of the disease, the patient’s personality, environment, objectives as well as clinical assessment, and must be regularly adjusted [34], [49], [50]. Some CPGs [36] provided a disclaimer for certain non-pharmacological recommendations, such as heat therapy which was not recommended for inflamed joints. Clinical algorithms may facilitate clinical decision making [30]–[32], [34], [44].

Often, physiotherapy and occupational therapy are classified as interventions, but in fact they are professions. Physiotherapists and occupational therapists provide a large spectrum of non-pharmacological interventions to patients with RA. Consequently more precision needs to be provided, especially regarding therapeutic interventions. The adoption of a PICOT [7] format to report the recommendation would be an asset for clinicians. P indicates the characteristics of the population for which the intervention is effective (e.g. stage of disease, disease duration etc…); I represents the characteristics of the intervention (e.g. dosage etc…); C is the comparator (placebo, control etc.); O indicates outcomes for which the intervention is effective; T is the time (short vs. long term effects) to apply the intervention in order to improve effectiveness or to learn the retention effect after stopping the intervention). Luqmani et al. [30] discussed the need for reporting the lasting impact of the effective intervention. The Ottawa Panel CPG [38]–[42] is the only one that used the PICOT format to frame its recommendations.

Almost all CPGs recommended or strongly recommended patient education, aerobic exercise, dynamic exercise and strengthening exercises. Further, majority of the CPGs recommended joint protection, splinting, multidisciplinary team approach and whole body exercises. Although therapeutic exercises to maintain joint range of motion and mobility were generally recommended by all the CPGs. However, there is a need for a more specific scientific basis of the benefits and potential harms related to types of exercises [44]. Exercise dose-response in relation to disease activity, joint symptoms, level of fatigue and quality of life need to be determined for the optimal management of RA [15], [47].

More precisions on dosage are also needed on the use of splints (e.g. static vs. dynamic/working exercise) [31], [41], patient education (e.g. group vs. individual; self-management) [31], [41] and thermotherapy (e.g. heat application vs. cryotherapy) [31] and needs to be categorized or more detailed in some CPGs. Operational definitions also need to be provided (e.g. hydrotherapy vs. balneotherapy vs. aquatics).

Most CPGs recognized that the management of RA must include pharmacological and non-pharmacological interventions [30]–[35], [37], [42]–[44]. Additionally, non-pharmacological approaches should also explore the consideration of combined interventions to be more representative of the clinical context. Primary RCTs that were used to build the CPG, examined the specific effect of monotherapy, whereas combined interventions are current practice in rehabilitation. These need to be explored to reflect current practices, as many interventions may be effective or more effective when used in combination, such as paraffin wax alone versus paraffin wax combined with exercise.

### Descriptive Analysis

The sample size of the included CPGs was too small to perform any additional analysis such as a student t test or ANOVA test and test for statistical significance of the improvement of CPGs over time. Based on figure S2 the overall quality of the CPG does not improve over time, many of the CPGs published earlier [39]–[40] were considered to be of higher quality CPGs compared to those published more recently. A rationale for why some of the earlier published CPGs had a much higher quality than the more recently published CPGs could be attributed to the methodology each guideline used. The Ottawa panel guidelines follows the Cochrane methodology when developing their guidelines and this rigorous evidence based method resulted in higher quality CPGs compared to the CPGs published more recently using another method of development.

### Limitations

The AGREE II instrument is updated versions of the initial AGREE I instrument [12]–[13]. AGREE II was available in 2009 with validation studies conducted in 2010 [12]–[13], [52]. Despite this fact, more recent CPGs published after this date [33], [38], [45] did not receive a high overall quality score and did not effectively address all the domains, especially for items in domain 5 (applicability) and domain 6 (editorial independence). Another limitation of the AGREE II is it evaluates only the quality of the reporting versus the quality of the development [48], [51]. As only the published and available documents were evaluated, the ratings for rigor of development were lower than they might have been if we had contacted the developers for the required documentation. We considered the scoring system (e.g. discrimination between a score of 4 or 5) and the subjectivity of interpreting the domain criteria as a limit to the AGREE II instrument. The 7-point scale used in AGREE II is based on the idea that if all the elements of a particular item are fully addressed, then it is given a score of seven for that particular item. Conversely, if none of the elements were present then it was given a score of one. An initial score of one (absence of information) can be considered a systematic error, because AGREE II does not consider “not applicable” a response according to its scoring system. Finally, there is a potential publication bias that may limit generalizability of our results as only CPGs published in English were selected.

## Conclusion

In summary, the literature search yielded 13 CPGs that targeted non-pharmacological interventions for RA. Six CPGs were found to be of high quality based on the AGREE II instrument and the non-pharmacological interventions that were recommended were: patient education/self-management, aerobic, dynamic and stretching exercises. The authors found that the recommendations presented in the CPGs provided insufficient information on the mode of delivery, dosage, intensity, frequency and duration. Future CPG developers should focus more on addressing all six AGREE II domains, in particular rigor of development and applicability to ensure the recommendations presented can easily be implemented in daily health care practice.

## Supporting Information

Figure S1 **Prisma flow diagram of included CPGs.**
(DOC)

Figure S2 **CPG Quality Over Time.**
(JPG)

Table S1 **Recommendations for the Management of Rheumatoid Arthritis.** ACR: American College of Rheumatology, BSR: British society of rheumatology; EULAR: The European League against rheumatism; NICE: National Institute for health and Clinical Excellence; PGrip: People Getting A Grip on Arthritis (www.arthritis.ca/PeopleGettingaGrip); SIGN: Scottish Intercollegiate Guidelines network Scottish Intercollegiate Guidelines network; NA: Not Assessed; TENS: Transcutaneous Electric Nerve Stimulation.
(DOCX)

Appendix S1 **Literature Search Strategy.**
(DOCX)

Appendix S2 **Selection criteria for clinical practice guidelines.**
(DOCX)

Appendix S3 **Raw data of the AGREE II items.**
(DOC)

Checklist S1 **Prisma 2009 Checklist.**
(DOC)
