# Supplementary material for: A Systematic Critical Appraisal of Non-Pharmacological Management of Rheumatoid Arthritis with Appraisal of Guidelines for Research and Evaluation II
Source: PLoS One. 2014 May 19;9(5):e95369. doi: 10.1371/journal.pone.0095369 (PMC4026323; doi:10.1371/journal.pone.0095369)
Supplement: Figure S1 — Prisma flow diagram of included CPGs. (DOC) [file pone.0095369.s001.doc]

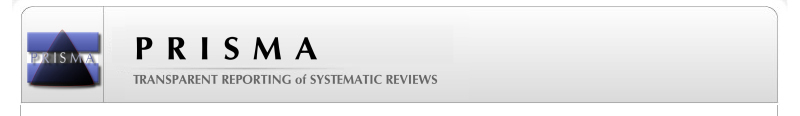
**Figure S1 PRISMA 2009 Flow Diagram**

**Screening**

**Included**

**Eligibility**

**Identification**

Records identified through database searching
(n=1136)

Additional records identified through other sources (hand search) (n=1)
(n =2)

Records after duplicates removed
(n=827)

Records screened
(n=40)

Records excluded based on abstract and title
(n=787)

Full-text articles assessed for eligibility
(n=18)

Full-text articles excluded, with reasons
(n=7):

- The guideline could not be retrieved (n=1)
- The journal was not found or article not indexed (n=1)
- The article was a summary (n=2)
- The guideline was published in 2000 (n=1)
- The article was an editorial (n=1)
- The article was a literature review (n=1)

Studies included in qualitative synthesis
(n=13)

Studies included in quantitative synthesis (meta-analysis)
(n=13)
