# Supplementary figures and images for: A Systematic Critical Appraisal of Non-Pharmacological Management of Rheumatoid Arthritis with Appraisal of Guidelines for Research and Evaluation II
Source: PLoS One. 2014 May 19;9(5):e95369. doi: 10.1371/journal.pone.0095369 (PMC4026323; doi:10.1371/journal.pone.0095369)

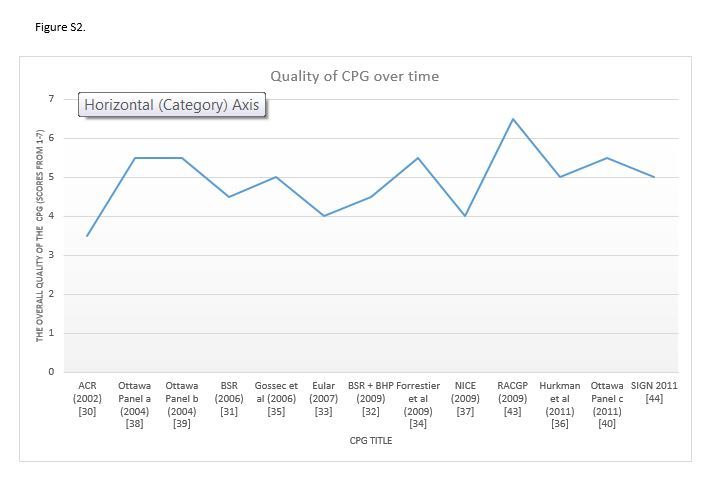

Supplement: Figure S2 — CPG Quality Over Time. (JPG) [file pone.0095369.s002.jpg]
