# Supplementary material for: A Systematic Critical Appraisal of Non-Pharmacological Management of Rheumatoid Arthritis with Appraisal of Guidelines for Research and Evaluation II
Source: PLoS One. 2014 May 19;9(5):e95369. doi: 10.1371/journal.pone.0095369 (PMC4026323; doi:10.1371/journal.pone.0095369)
Supplement: Table S1 — Recommendations for the Management of Rheumatoid Arthritis. ACR: American College of Rheumatology, BSR: British society of rheumatology; EULAR: The European League against rheumatism; NICE: National Institute for health and Clinical Excellence; PGrip: People Getting A Grip on Arthritis (www.arthritis.ca/PeopleGettingaGrip); SIGN: Scottish Intercollegiate Guidelines network Scottish Intercollegiate Guidelines network; NA: Not Assessed; TENS: Transcutaneous Electric Nerve Stimulation. (DOCX) [file pone.0095369.s003.docx]

| **Intervention** | ACR | BSR | BSR & BHP | Eular | Forrestier | Gossec | Hurkmans | NICE | Ottawa Panel | RACGP | SIGN |
| --- | --- | --- | --- | --- | --- | --- | --- | --- | --- | --- | --- |
|  |  |  |  |  | et al. | et al. | et al. 2011b |  |  |  |  |
|  | [29] | [30] | [31] | [32] | [33] | [34] | [35] | [36]] | [37-41] | [42] | [43] |
| **Electrotherapy** | | | | | | | | | | | |
| Electrical stimulation of muscle | NA | NA | NA | NA | NA | NA | Insufficient evidence | NA | Insufficient evidence | NA | NA |
| Low intensity laser therapy | NA | NA | NA | Insufficient evidence | NA | NA | Insufficient evidence | NA | Strongly recommended | Insufficient evidence | Insufficient evidence |
| TENS (high frequency) | NA | Weak Evidence | NA | Insufficient evidence | NA | NA | Insufficient evidence | Recommended | Insufficient evidence | Insufficient evidence | Insufficient evidence |
| TENS (low frequency) | NA | Weak Evidence | NA | Insufficient evidence | NA | NA | Insufficient evidence | Recommended | Strongly recommended | Insufficient evidence | Insufficient evidence |
| Therapeutic ultrasound | NA | NA | NA | Insufficient evidence | NA | NA | Insufficient evidence | NA | Strongly recommended | Insufficient evidence | Weak evidence |
| **Other Interventions** | | | | | | | | | | | |
| Acupuncture | NA | NA | NA | NA | Insufficient Evidence | NA | NA | NA | NA | Insufficient evidence | NA |
| Assistive devices | NA | Weak evidence | Weak evidence | Insufficient evidence | Weak evidence | NA | NA | NA | Insufficient evidence | Weak evidence | NA |
| Balneotherapy | NA | Weak evidence | NA | Recommended | Weak evidence | NA | Insufficient evidence | NA | NA | NA | NA |
| Complementary and alternative therapies | NA | Weak evidence | NA | Insufficient Evidence | Insufficient Evidence | NA | Insufficient Evidence | NA | NA | Recommended | Insufficient Evidence |
| Energy Conservation | Recommended | NA | NA | NA | NA | NA | NA | NA | Insufficient evidence | NA | NA |
| Foot Orthoses and insoles | NA | Recommended | NA | Insufficient evidence | Weak evidence | Recommended | NA | NA | Recommended | Weak evidence | Recommended |
| Heat/Cryotherapy | NA | Weak evidence | NA | Insufficient evidence | NA | NA | Insufficient evidence | NA | Insufficient evidence | Insufficient evidence | Insufficient evidence |
| Hydrotherapy | NA | Weak evidence | NA | Recommended | NA | NA | NA | NA | NA | NA | NA |
| Joint Protection | Recommended | Weak evidence | Strongly Recommended |  |  | Insufficient Evidence | NA |  | Strongly Recommended | Recommended | Insufficient evidence |
| Paraffin wax application | NA | Weak evidence | NA | NA | NA | NA | NA | Recommended | Insufficient evidence | NA | NA |
| Paraffin wax application + exercises | NA | NA | NA | NA | NA | NA | NA | NA | Strongly Recommended | NA | NA |
| Patient Education/self-management | Recommended | Recommended | Recommended | Recommended | Recommended | Insufficient Evidence | Recommended | Recommended | Strongly Recommended | Weak evidence | Insufficient evidence |
| Splinting | NA | Weak evidence | Strongly Recommended | Insufficient evidence | Insufficient evidence | NA | NA | NA | Recommended | Insufficient evidence | Recommended |
| **Team Approach** | | | | | | | | | | | |
| Multidisciplinary Team Approach | Recommended | Recommended | NA | Recommended | Recommended | NA | NA | Recommended | NA | NA | Recommended |
| Dietician | Weak evidence | NA | NA | Recommended | NA | NA | NA | Recommended | NA | NA | NA |
| Medical | Recommended | Recommended | Recommended | Recommended | Recommended | Recommended | NA | Recommended | NA | Recommended | Recommended |
| Nursing | Recommended | Weak evidence | NA | NA | NA | NA | NA | NA | NA | NA | NA |
| Occupational Therapy | Recommended | Recommended | Weak evidence | Recommended | NA | NA | NA | Recommended | NA | Recommended | Recommended |
| Podiatrist/Chiropodist | NA | Recommended | NA | NA | Insufficient evidence | NA | NA | Recommended | NA | Recommended | Weak evidence |
| Pharmacist | NA | NA | NA | NA | NA | NA | NA | NA | NA | NA | NA |
| Physiotherapy | Recommended | Recommended | NA | NA | Weak evidence | NA | Recommended | Recommended | Recommended | NA | Recommended |
| Psychology | Recommended | Recommended | Recommended | NA | NA | NA | NA | Recommended | NA | Weak evidence | NA |
| Social Work | Recommended | NA | NA | NA | NA | NA | NA | NA | NA | NA | NA |
| **Therapeutic Exercises** | | | | | | | | | | | |
| Aquatics | NA | NA | NA | NA | NA | Recommended | Insufficient evidence | NA | Recommended | NA | NA |
| Aerobic Exercises (AE) | Recommended | Recommended | Recommended | Recommended | Recommended | Recommended | Strongly Recommended | Recommended | Recommended | Weak evidence | Strongly Recommended |
| Low intensity Exercises | NA | NA | NA | NA | NA | NA | NA | NA | Strongly Recommended | NA | Strongly Recommended |
| Dynamic Exercises | Recommended | Strongly Recommended | Strongly Recommended | Strongly Recommended | Strongly Recommended | Recommended | Strongly Recommended | Strongly Recommended | Strongly Recommended | Recommended | Strongly Recommended |
| Stretching | NA | NA | NA | NA | NA | NA | NA | Recommended | NA | NA | NA |
| Strengthening Exercise (SE) | Recommended | Recommended | Recommended | Recommended | Recommended | Recommended | Strongly Recommended | Recommended | Recommended | Weak evidence | Strongly Recommended |
| Whole body exercises (Tai Chi and Yoga) | Recommended | Recommended | Recommended | Recommended | NA | Recommended | NA | Recommended | Recommended | Weak evidence | Strongly Recommended |
| **Weight Management** | | | | | | | | | | | |
| Control of weight | NA | NA | NA | Insufficient evidence | NA | NA | NA | NA | NA | Weak evidence | Recommended |
| Diet | NA | NA | NA | Insufficient evidence | Insufficient evidence | NA | NA | Weak evidence | NA | Strongly Recommended | Recommended |
| Diet supplement | NA | NA | NA | Insufficient evidence | NA | Not Recommended | NA | NA | NA | Strongly Recommended | Weak evidence |
| Diet and Physical Activity | NA | NA | NA | Insufficient evidence | NA | NA | NA | NA | NA | Recommended | NA |

**Table S1 Recommendations for the Management of RA.**

**Table S1. Recommendations for the Management of Osteoarthritis**

ACR: American College of Rheumatology, BSR: British society of rheumatology; EULAR: The European League against rheumatism; NICE: National Institute for health and Clinical Excellence; PGrip: People Getting A Grip on Arthritis (www.arthritis.ca/PeopleGettingaGrip); SIGN: Scottish Intercollegiate Guidelines network Scottish Intercollegiate Guidelines network; NA: Not Assessed; TENS: Transcutaneous Electric Nerve Stimulation.
