# Supplementary material for: A Systematic Critical Appraisal of Non-Pharmacological Management of Rheumatoid Arthritis with Appraisal of Guidelines for Research and Evaluation II
Source: PLoS One. 2014 May 19;9(5):e95369. doi: 10.1371/journal.pone.0095369 (PMC4026323; doi:10.1371/journal.pone.0095369)
Supplement: Appendix S2 — Selection criteria for clinical practice guidelines. (DOCX) [file pone.0095369.s005.docx]

Appendix S2. Selection criteria for clinical practice guidelines.

| **The Inclusion Criteria** | **Rationale for the Inclusion and Exclusion Criteria** | **The Exclusion Criteria.** |
| --- | --- | --- |
| 1. CPGs that are published in a peer reviewed journal. | 1. Accessibility of web based CPGs is more limited after a certain amount of time. | 1. CPGs published only on the web. |
| 1. Authors must be identified in the CPGs. | 1. Indication of the authors’ name must be clear in each CPG. | 1. CPGs with a professional group/association as the author |
| 1. A reference list must be included in the CPGs. | 1. Scientific data must be used in the CPG and thus references must be provided. | 1. CPGs with no reference list |
| 1. A grading system for evaluating the level of evidence for the recommendations must be in each CPG. | 1. Recommendations given in the CPG must be based on the body of evidence pertaining to the research question. | 1. CPGs lacking a grading system for the recommendations. |
| 1. Systematic reviews clearly used in the CPG. | 1. Publication biases are more likely to be avoided with systematic reviews as they provide recommendations that are less biased. | 1. CPGs without clear indication they are based on systematic reviews |
| 1. CPGs have to be written in English. | 1. Understanding the guideline and AGREE II content requires the CPGs to be written in English. | 1. CPGs written in any other language than English were excluded. |
| 1. Current CPGs (2001-2012) are included and they have to mention a release date. | 1. Including a release date ensured only recent CPGs were included. | 1. CPGs published before the year 2000. |
| 1. Participants in the CPGs must be >18 years old. | 1. Recommendations for paediatrics are not sufficiently specific. | 1. CPGs with participants <18 years old. |
| 1. A comparative controlled study examining the effectiveness of specific non-pharmacological interventions in rheumatology must be in the CPG. | 1. Publication of guidelines by professional associations may be on ethics or practice standards instead of physical rehabilitation interventions. CPGs selected referred to physical interventions. | 1. CPGs focusing only on pharmaceutical or surgical interventions. |
| 1. The CPGs must be pertinent to rheumatoid arthritis (RA) in the extremities of the body. | 1. RA is one of the most prevalent diseases in Rheumatology. RA in the spine may require different interventions due to the presence of neurological conditions. | 1. CPGs that included other rheumatologic conditions. |
