# Supplementary material for: A Systematic Critical Appraisal of Non-Pharmacological Management of Rheumatoid Arthritis with Appraisal of Guidelines for Research and Evaluation II
Source: PLoS One. 2014 May 19;9(5):e95369. doi: 10.1371/journal.pone.0095369 (PMC4026323; doi:10.1371/journal.pone.0095369)
Supplement: Appendix S3 — Raw data of the AGREE II items. (DOC) [file pone.0095369.s006.doc]

| AGREE II items | ACR  2009 | | BSR  2006 | | BSR  2009 | | EULAR  2007 | | Forestier et al.  2009 | | Gossec et al.  2006 | | Hurkmans et al | | NICE  2009 | | Ottawa  Panel  Guidelines 2004a & b; 2011 | | RACGP  2009 | | Sign 2011 | |
| --- | --- | --- | --- | --- | --- | --- | --- | --- | --- | --- | --- | --- | --- | --- | --- | --- | --- | --- | --- | --- | --- | --- |
|  | Domain 1. Scope and Purpose | | | | | | | | | | | | | | | | | | | | | |
| 1 | 2 | 2 | 5 | 7 | 7 | 7 | 2 | 2 | 7 | 7 | 7 | 7 | 6 | 5 | 6 | 6 | 7 | 7 | 7 | 7 | 6 | 7 |
| 2 | 2 | 2 | 6 | 6 | 6 | 6 | 2 | 2 | 6 | 7 | 6 | 6 | 5 | 5 | 5 | 6 | 7 | 7 | 6 | 7 | 5 | 5 |
| 3 | 2 | 2 | 6 | 6 | 6 | 6 | 2 | 2 | 5 | 5 | 6 | 6 | 1 | 1 | 5 | 5 | 6 | 7 | 7 | 7 | 6 | 6 |
|  | Domain 2. Stakeholder Involvement 7 | | | | | | | | | | | | | | | | | | | | | |
| 4 | 3 | 4 | 7 | 7 | 7 | 7 | 6 | 7 | 7 | 7 | 7 | 7 | 7 | 7 | 7 | 7 | 6 | 6 | 6 | 7 | 7 | 7 |
| 5 | 2 | 2 | 4 | 4 | 4 | 4 | 1 | 1 | 3 | 2 | 1 | 1 | 5 | 6 | 6 | 6 | 4 | 4 | 5 | 6 | 6 | 6 |
| 6 | 2 | 2 | 5 | 6 | 5 | 6 | 2 | 2 | 5 | 6 | 7 | 6 | 5 | 7 | 5 | 7 | 6 | 6 | 6 | 7 | 7 | 7 |
|  | Domain 3. Rigour of Development | | | | | | | | | | | | | | | | | | | | | |
| 7 | 1 | 1 | 2 | 2 | 2 | 2 | 2 | 2 | 7 | 7 | 6 | 7 | 7 | 6 | 5 | 7 | 6 | 7 | 7 | 7 | 6 | 6 |
| 8 | 1 | 1 | 3 | 3 | 3 | 3 | 3 | 3 | 5 | 6 | 4 | 4 | 6 | 5 | 5 | 6 | 7 | 7 | 7 | 6 | 1 | 1 |
| 9 | 1 | 1 | 2 | 2 | 2 | 2 | 4 | 4 | 4 | 4 | 4 | 4 | 5 | 6 | 4 | 3 | 7 | 7 | 2 | 3 | 3 | 1 |
| 10 | 2 | 2 | 4 | 2 | 4 | 3 | 7 | 7 | 6 | 7 | 7 | 7 | 4 | 6 | 6 | 7 | 4 | 4 | 6 | 7 | 5 | 5 |
| 11 | 3 | 3 | 3 | 3 | 4 | 4 | 3 | 2 | 4 | 5 | 3 | 3 | 5 | 7 | 2 | 2 | 5 | 5 | 7 | 7 | 2 | 2 |
| 12 | 3 | 2 | 3 | 3 | 3 | 2 | 4 | 3 | 7 | 7 | 4 | 6 | 7 | 7 | 5 | 5 | 5 | 5 | 7 | 7 | 5 | 6 |
| 13 | 2 | 2 | 5 | 5 | 4 | 3 | 5 | 5 | 6 | 7 | 6 | 5 | 5 | 4 | 3 | 2 | 3 | 4 | 7 | 7 | 7 | 5 |
| 14 | 1 | 1 | 4 | 3 | 3 | 3 | 2 | 2 | 1 | 1 | 1 | 1 | 1 | 1 | 2 | 2 | 1 | 1 | 4 | 6 | 7 | 7 |
|  | Domain 4. Clarity of Presentation | | | | | | | | | | | | | | | | | | | | | |
| 15 | 1 | 1 | 6 | 7 | 6 | 5 | 2 | 2 | 7 | 7 | 3 | 5 | 6 | 6 | 3 | 3 | 5 | 5 | 7 | 7 | 6 | 7 |
| 16 | 2 | 1 | 5 | 6 | 5 | 3 | 1 | 1 | 7 | 7 | 1 | 1 | 7 | 7 | 3 | 3 | 5 | 5 | 7 | 7 | 7 | 7 |
| 17 | 3 | 2 | 6 | 6 | 4 | 7 | 3 | 2 | 7 | 7 | 5 | 5 | 7 | 7 | 5 | 3 | 4 | 4 | 7 | 7 | 7 | 7 |
|  | Domain 5. Applicability | | | | | | | | | | | | | | | | | | | | | |
| 18 | 1 | 1 | 2 | 2 | 2 | 2 | 1 | 1 | 4 | 5 | 1 | 1 | 1 | 1 | 1 | 1 | 4 | 2 | 1 | 1 | 1 | 1 |
| 19 | 1 | 1 | 5 | 5 | 2 | 1 | 1 | 1 | 4 | 4 | 1 | 1 | 2 | 2 | 4 | 4 | 1 | 1 | 5 | 5 | 5 | 5 |
| 20 | 5 | 5 | 2 | 2 | 1 | 2 | 1 | 1 | 3 | 3 | 1 | 1 | 1 | 1 | 2 | 2 | 2 | 1 | 1 | 1 | 1 | 2 |
| 21 | 1 | 1 | 7 | 7 | 1 | 1 | 1 | 1 | 4 | 3 | 1 | 1 | 1 | 1 | 1 | 1 | 1 | 1 | 1 | 1 | 5 | 5 |
|  | Domain 6. Editorial Independence | | | | | | | | | | | | | | | | | | | | | |
| 22 | 1 | 1 | 2 | 1 | 2 | 1 | 1 | 1 | 1 | 1 | 3 | 4 | 2 | 2 | 7 | 7 | 5 | 5 | 4 | 6 | 5 | 5 |
| 23 | 5 | 6 | 5 | 5 | 6 | 6 | 1 | 1 | 7 | 7 | 1 | 1 | 1 | 1 | 6 | 6 | 2 | 2 | 1 | 1 | 4 | 3 |
|  | Overall Assessment | | | | | | | | | | | | | | | | | | | | | |
| A^1^ | 4 | 4 | 5 | 5 | 5 | 4 | 5 | 3 | 5 | 6 | 5 | 5 | 5 | 5 | 4 | 4 | 5 | 6 | 6 | 7 | 6 | 4 |
| B^2^ | M | M | M | M | M | M | N | N | Y | Y | M | M | Y | Y | M | M | Y | Y | Y | Y | M | M |

**Appendix S3 Raw data of the AGREE II items**

ACR: American College of Rheumatology , BSR: British society of rheumatology;; EULAR : The European League against rheumatism; NICE : National Institute for health and Clinical Excellence; SIGN: Scottish Intercollegiate Guidelines network Scottish Intercollegiate Guidelines network**^1^ A: Rate the overall quality of this guideline. ^2^B: I would recommend this guideline for use** **Y: Yes;**  **M: Yes, with modification; N: No**
